# Clinical Perspective on Passive Audio Vocal Measurement in the Evaluation of Selective Mutism

**DOI:** 10.3389/fpsyt.2018.00443

**Published:** 2018-09-21

**Authors:** Helen Y. Xu, Jacob Stroud, Renee K. Jozanovic, Jon Clucas, Jake Jungwoo Son, Bonhwang Koo, Juliet Schwarz, Arno Klein, Rachel Busman, Michael P. Milham

**Affiliations:** ^1^Sidney Kimmel Medical College, Thomas Jefferson University, Philadelphia, PA, United States; ^2^Northeastern University, Boston, MA, United States; ^3^Child Mind Institute, New York, NY, United States

**Keywords:** selective mutism, anxiety disorders, objective measures, wearable sensors, wearable devices

## Abstract

Selective Mutism (SM) is an anxiety disorder often diagnosed in early childhood and characterized by persistent failure to speak in certain social situations but not others. Diagnosing SM and monitoring treatment response can be quite complex, due in part to changing definitions of and scarcity of research about the disorder. Subjective self-reports and parent/teacher interviews can complicate SM diagnosis and therapy, given that similar speech problems of etiologically heterogeneous origin can be attributed to SM. The present perspective discusses the potential for passive audio capture to help overcome psychiatry's current lack of objective and quantifiable assessments in the context of SM. We present supportive evidence from two pilot studies indicating the feasibility of using a digital wearable device to quantify child vocalization features affected by SM. We also highlight comparative analyses of passive audio capture and its potential to enhance diagnostic characterizations for SM, as well as possible limitations of such technologies.

## Introduction

Selective Mutism (SM) is an anxiety disorder characterized by persistent failure to speak in certain social situations but not others. SM is often diagnosed in early childhood when children are expected to start engaging in typical social interactions (1). Children with SM are typically comfortable speaking in their home environment yet tend to struggle when challenged with novel social situations—particularly the school environment (2). SM-related symptoms can present long-term difficulties for children in developing social communication skills, in performing at school and in engaging with peers or others (3).

There exists a long history of changing conceptualizations of SM. Symptoms relating to SM were described as early as the late 1800s, when the disorder was referred to as “aphasia voluntaria” (4). The disorder was first captured in the psychiatric nosology of the Diagnostic Statistical Manual (DSM) in its third edition in 1980, wherein “elective mutism” was formally introduced. The criteria and interpretation of the disorder focused on a child's *refusal* to speak, emphasizing beliefs that the disorder was rooted in defiance or trauma (5). As the field increasingly questioned the volitional nature of the disorder, the DSM-IV made two major changes: (1) the key diagnostic criterion was changed from a “*refusal* to speak” to a “*failure*,” and (2) the name was changed to *selective* mutism. These changes deemphasized unwillingness and oppositionality and moved away from strictly psychosocial conceptualizations of SM. SM remained classified under “Other Disorders of Infancy, Childhood, and Adolescence” (6). Over the last decade, a growing consensus has pointed toward roots of SM in anxiety, (and in 2013, the DSM-V reclassified SM from childhood disorders to anxiety disorders (2, 7).

While SM was traditionally considered a relatively uncommon disorder, recent estimates suggest a prevalence of 0.47–1.0% of the population (2, 3, 8); the increased estimates are thought to reflect a growing awareness of the disorder and prior misdiagnoses (e.g., autism, communication disorder, PTSD, or just “shyness”) (9, 10). In addition, SM tends to be more prevalent in girls than boys, with some literature studies suggesting as high as 2:1 female to male ratio (11, 12). Although less frequently diagnosed beyond childhood, SM can affect adults and has been associated with symptoms such as social anxiety and low self-confidence; these can appear as adult-onset or as symptoms extending from childhood (13, 14).

Although interest in SM is growing, research is relatively limited in comparison to research on other disorders of similar prevalence or severity (15, 16). To date, much of SM research has consisted of case studies or intervention trials with small samples, making replication and generalization difficult.

SM has historically been considered difficult to treat, with residual symptoms often persisting long after treatment (17). As a condition of likely multifactorial origin, possible treatments for SM span a range of modalities (18, 19). Behavioral interventions may include contingency management, shaping, stimulus fading, and systematic desensitization (20–23). Cognitive behavioral therapy has also demonstrated efficacy in this population, consistent with conceptualizations of SM as an anxiety disorder (24, 25). Pharmacological approaches to SM tend to prioritize selective serotonin reuptake inhibitors, again supporting anxiety-focused conceptualizations (17, 26, 27). Technology-based methods, such as using iPads for modeling or feedback, or augmentative and alternative communication (AAC) methods (e.g., text-to-speech systems) have been employed in SM treatment as well (28, 29).

Unfortunately, there are few standardized and objective tools for quantification of symptoms before and after treatment. Most assessments rely on subjective report and single raters, creating the potential for biases, thereby limiting their expected utility. In a review by Kratochwill, it was recommended that “direct measures of speech […and] physiological measures seem especially relevant in research and treatment of mutism.” (2014, 130–132). Tools such as the ADIS, a semi-structured interview for anxiety disorders that has an SM-dedicated module can be used to gather data for diagnosis (30, 31). Evaluations to rule out alternative diagnoses may include speech and language, oral-motor, and hearing assessments. Some providers carry out live behavioral observation sessions to gather data regarding how an affected child interacts with different individuals, including the parent (32). Finally, the Selective Mutism Questionnaire (SMQ) and the corresponding School Speech Questionnaire are commonly used to help quantify symptom severity (33).

In this relative void of high quality assessment tools, passive audio/vocal capture is rapidly emerging as a promising assessment modality for psychiatry and for SM. A benefit of passive, unintrusive devices for children with SM is maximizing their comfort, particularly because these children often become more anxious in new settings with new people. Passive audio/vocal capture is growing increasingly sophisticated, with new analytic platforms for automated extraction of features that can be used to predict states and behaviors (e.g., anxiety, depression, suicidality). Existing wearable technologies in consumer and research domains have already been successfully applied to monitor a range of behaviors and responses, including sleep, diet, electrodermal activity, and heart rate (34, 35). The successes of devices such as the Fitbit and Apple Watch have helped to increase public acceptance of, and sometimes reliance on, wearable devices. Sensors for minimally intrusive audio capture have been employed in areas including stress research (36) and nursing home monitoring (37). LENA, a device and software allowing for passive measurement of vocalization counts, vocal volume, and other conversational measures in children LENA (38), has been employed in recent speech studies, including studies of children with ASD and of bilingual children (39, 40). Simple passive audio tools applied to SM could provide objective measures to better characterize the disorder without relying on complex analytics, burdensome devices (41, 42), or multiple biased reports.

## A test of feasibility: the LENA device

Here we present findings from two initial tests of feasibility for the use of passive vocal recording to assess individuals with SM. We made use of LENA digital language processors (DLPs), the benefits of which include: (1) size smaller than a deck of cards, (2) availability of t-shirts designed to house a DLP in a chest pocket, (3) ready availability of automated feature extraction software, and (4) ability to record and parse speech from the child and nearby speakers.

In both test applications, participants were provided a LENA shirt and DLP, which they decorated with name tags and stickers in an effort to acclimate the children to the shirts.

## Test 1: brave buddies

Brave Buddies is an intensive 1-week SM treatment program at the Child Mind Institute that draws from a number of previously established behavioral techniques (i.e., adapted parent-child interaction therapy, group therapy, and parent training). Our primary goals were to assess: (1) the ability of children with SM to tolerate wearing a LENA DLP for an extended period of time and (2) the ability of the DLP to detect relevant changes during the course of the intervention.

### Methods

#### Participants

Twelve of 36 patients enrolled in Brave Buddies agreed to simultaneously participate in the LENA research study (9 female, 3 male; ages 5–8).

#### Design

**Figure 1:**
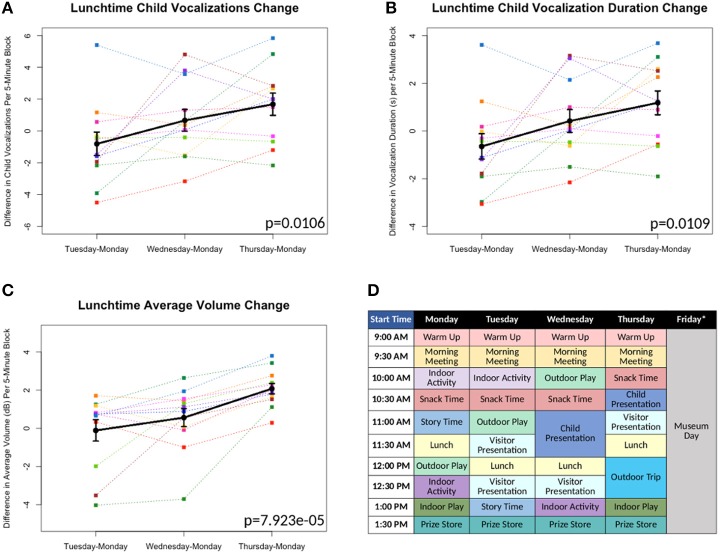
**(A-C)** Differences in measures as compared to Monday's baseline values plotted^*^. Each color line represents a different individual participant. Means with standard error bars plotted in black. **(D)** Sample schedule for Brave Buddies week, showing various activities. ^*^Friday data excluded from analyses, as described in Results.

Brave Buddies took place in a classroom-like setting and was structured like a typical school day, with each day divided into activity blocks (see Figure 1D). Patients were separated into three age-based groups of 12 children each, with one LENA study participant in the ages 4–5 years group, five in the 5–6 years group and six in the 6–8 years group. Each group had its own room and dedicated counselors trained in behavioral techniques. Each child was also paired with a counselor who accompanied the child throughout each day's activities and regularly prompted the child to answer questions and to vocalize. Throughout the treatment program, research staff accompanied each of the three groups, noting start and end times of each activity block, as well as information about deviations and factors that might affect the quality of the recordings.

#### Feature extraction

LENA Pro, a software companion to the DLPs, provides numerous measures from the collected data (43, 44); from these measures, we focused on a few measures of interest:

- *Vocalization counts:* instances of speech-related sounds separated by at least 300 ms of silence, produced by the child wearing the DLP.
- *Vocalization duration:* combined duration in seconds for all speech by the child per 5-min block.
- *Average volume:* average decibel level per 5-min block.
- *Conversational turns:* distinct pairings of vocalizations between an adult and the child, which occur within 5 s of each other. In other words, if either the child or adult responds to the other within 5 s, that is considered one turn.

### Results

All 12 children were able to complete the 5-day assessment of the LENA DLP without any significant difficulties related to wearing the device. Brave Buddies data from Friday, during which the children spent the day visiting a museum, were excluded due to a divergent setting and structure compared to previous days. From Monday–Thursday, we found that the most vocalizations occurred during the Outdoor Play activity as compared to other activities. In comparing data across days, we focused on Lunchtime, an activity that allowed for open, freeform vocalizing and was consistent across days. Using multivariate repeated measures ANOVA (scripts used available at https://github.com/ChildMindInstitute/LENA_BB_CPP_analysis/tree/master/BB), we found significant increases across the days in Lunchtime vocalization count (*p* = 0.0106), vocalization duration (*p* = 0.0109), and average volume (*p* = 7.923e-05) per 5-min block (see Figures 1A–C); insignificant ANOVA results and no upward trends were observed in other activity blocks.

### Discussion

Multiple detectable vocal properties exhibited significant improvements across the 4 days included in our examination (Monday–Thursday), though only during Lunchtime. The specific sensitivity of Lunchtime to changes in behavior may be informative; specifically, this was among the least structured and directed of activities, with less feedback and interaction from clinical staff. This finding suggests that there are limitations to simply applying a DLP to an ongoing intervention that does not specifically facilitate assessment of freeform speech. Looking forward, introduction of more such periods could increase the utility of passive audio capture in structured clinical intervention programs such as Brave Buddies.

There were two additional limitations of using LENA for tracking progress during Brave Buddies. First, despite its structure, the program involved numerous variables that were difficult to control from a research standpoint, such as lack of experimental controls, minimal freeform speech, and lack of consistency in treatment applications. For example, the school-type activities in Brave Buddies were structured so that children would not be continually speaking and treatment was based on individual needs and severity, conditions that varied across children. Second, participants in Brave Buddies were selectively biased toward less severe cases of SM who would be able to tolerate an unfamiliar group setting. Whether LENA and related wearables will be feasible with more severe populations remains unclear.

## Test 2: controlled play paradigm

We conducted a more controlled assessment of the LENA DLP in which children were assessed one at a time and interactions more regulated. Specifically, we assessed children wearing a LENA while they were playing with their parent in an observation room in a design based on Parent-Child Interaction Therapy (45). Because a foreign environment alone may not be enough to evoke SM symptoms, we also varied whether a male experimenter was present and if present, whether he interacted with the child. The Controlled Play Paradigm was intended to test whether audio features extracted by the LENA software could differentiate children with SM from controls and to investigate correspondence between these features and established questionnaires (i.e., SMQ).

### Methods

#### Participants

Twelve children diagnosed with SM ages 5–8 (9 female, 3 male) participated, including 7 who also participated in Test 1 (Brave Buddies). Twelve age-matched controls without any reported diagnoses were recruited from the community, ages 5–8 (7 female, 5 male).

#### Design

At the start of the timed study, the child and parent were left alone in a room filled with various toys (e.g., blocks, toy animals, etc.). Research staff observed from another room via a one-way mirror. Speakers streamed audio into the staff observation room, and video was recorded with a view of the child and parent. After setup, video recording, and LENA recording were started simultaneously, with both recording 5 blocks of 10 min each. Video was used as a supplement to the LENA device and was not meant as a primary source of data. Human raters also coded data from the video as validation; their counts were compared to LENA counts and Pearson's r was calculated as 0.734 (child vocalizations) and 0.737 (conversational turns). The parent or guardian also completed questionnaires, including the SMQ, which assesses child vocalizations in different settings, as well as interference and distress.

Three block types were included in an alternating block design (A-B-A-C-A). In Block A (no stranger), the parent was instructed to play with their child alone and to ask their child questions. Block B (stranger without interaction) introduced a male member of the research staff who had not yet interacted with the child as the “stranger.” He entered the room, told the parent and child, “I am going to do some work over here,” and sat in a corner of the room without further interaction. In Block C (stranger with interaction), the same “stranger” returned to the room, sat next to the parent and child and asked, “It looks like you're having fun. Can I play with you?” The stranger engaged directly with the child, playing and asking questions (at least 2 per minute, often more). The parent was instructed to allow the stranger be the primary person asking questions during this block and to refrain from “saving” the child by answering questions intended for the child if the child failed to answer. During all blocks, the parent and stranger each wore an earpiece connected to a walkie talkie, through which observing research staff communicated.

## Results

**Figure 2:**
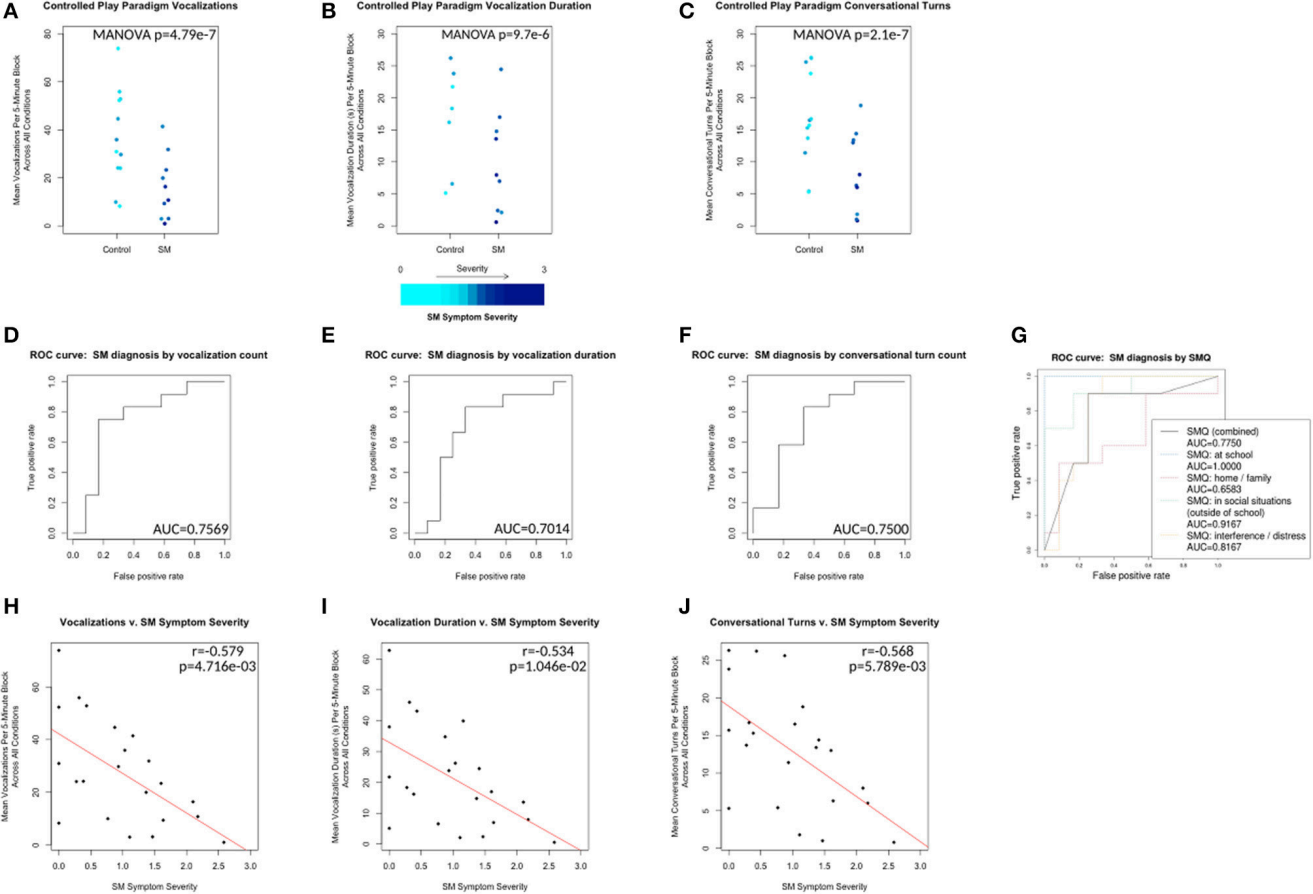
Control vs. SM groups plotted with respect to mean vocalization counts, mean vocalization durations, and mean conversational turn counts across all conditions (A_1_, B, A_2_, C and A_3_ collapsed). Plotted points color scaled to the individual's SM Symptom Severity score. **(D–F)** ROC curves for leave-one-out cross-validation of generalized linear models predicting control v. SM group membership from each of the same measures. **(G)** ROC curves for the same analysis of SMQ scores (combined and subscale) v. SM group membership. **(H–J)** Correlations plotted for same measures v. SM Symptom Severity for all 24 individuals. Line of best fit plotted in red.

The data were divided by group (Control v. SM) and by condition (block A v. B v. C). Multivariate ANOVA showed no significant main effect of condition or interaction effect between group and condition for any measures. However, the main effect of group was significant for vocalizations (*p* = 4.79e−07), vocalization duration (*p* = 9.7e−06), and conversational turns (*p* = 2.1e−07) (see Figures 2A–C). A leave-one-out cross-validation of a generalized linear model predicting SM diagnosis from each of these measures resulted in an area of > 0.7 under the receiver operating characteristic curve for each model, with most SMQ scores performing only slightly better (see Figures 2D–H).

SM Symptom Severity is a measure calculated based on SMQ responses from participants' parents. SMQ Interference/Distress subscores (ranging from 0 to 18) were scaled and inverted to match the other subscores (ranging from 3 to 0) by this formula: *interference_score_scaled* = *3* − *(interference_score* ÷ *6)*. The SM Symptom Severity was then calculated as 3 minus the mean score of the resulting 4 subscales (Home/Family, Social Situations, School and inverted Interference/Distress), representing an approximation of parent-reported SM-related symptom severity, with higher scores indicating increased severity. SM Symptom Severity was significantly negatively correlated with vocalizations (*r* = −0.579, *p* = 4.716e−03), vocalization duration (*r* = −0.534, *p* = 1.046e−02) and conversational turns (*r* = −0.568, *p* = 5.789e−03) (see Figures 2H–J).

## Discussion

Within the controlled environment, we found the LENA DLP with LENA software could be used to detect between-group differences in various measures of vocalization. In each of the three scenarios, children with SM and control groups differed in mean vocalization counts per 5-min block. A statistically significant linear relationship was demonstrated between SM Symptom Severity (calculated from SMQ responses) and each of three outcome measures extracted by the LENA software (i.e., vocalization count, duration and conversational turns). Thus, the LENA measures appear to be sensitive to SM-related changes in child vocalization, a promising step forward for future use in clinical populations.

## Conclusion

Selective Mutism is an understudied anxiety disorder that would benefit from objective measures to characterize the heterogeneity of symptoms and treatment outcomes. This study indicates that the extraction of features from passive audio can be informative for SM research.

The LENA device is appealing for assessment of clinical populations, such as SM patients due to its availability and automatic processing; however, the device presents specific limitations for use with these populations. LENA was developed for very young children, ages 0–4 years (46), and though our work indicates its potential for older participants, those populations are not the developers' focus. The LENA is also closed source and proprietary, meaning that its algorithms are unknown and immutable and we cannot know if our recordings are adequate for calibration. Lastly, the LENA is capable of recording *successful* vocalizations, but may not be able to detect unsuccessful or very low-volume vocalization attempts.

Moving forward, we will refine our experimental design based on lessons learned in this initial work, consider alternate or additional audio analysis options (47, 48) and develop more practical ways to use the LENA device for SM populations. The stimuli provided in each of these experiments did not provoke significant symptomatic behaviors from our participants; as such, future work may include more provocative stimuli (e.g., having a stranger offer a snack to probe for comorbid dysphagia).

As a behaviorally defined condition, SM appears to be derived from various heterogeneous factors (49), and “given the complexity of the phenomenon labeled 'selective mutism,' it appears that multiple measures and their degree of correspondence are necessary” [(50), p. 132]. Passive audio tools can provide multiple objective measures to better characterize SM and provide consistent feedback, empowering children and caregivers to better understand its etiology, to diagnose, and to treat SM in the future.

## Ethics statement

This study was carried out in accordance with the recommendations of the Chesapeake IRB with written informed consent from all subjects. All subjects gave written informed consent in accordance with the Declaration of Helsinki. The protocol was approved by the Chesapeake IRB.

## Author contributions

HX, JSt, RJ, RB, and MM contributed to the conception and design of the study. JSc contributed to participant recruitment. HX, JSt, RJ, and BK acquired data and obtained consent from all participants. HX, JSt, JC, JJS, and AK performed the statistical analysis. MM, HX, JSt, JJS, and JC wrote the manuscript. All authors contributed to manuscript revision, read and approved the submitted version.

### Conflict of interest statement

The authors declare that the research was conducted in the absence of any commercial or financial relationships that could be construed as a potential conflict of interest.
